# Supplementary material for: The relation between Self-Control, Need for Cognition and Action Orientation in secondary school students: A conceptual replication study
Source: PLoS One. 2023 Jun 9;18(6):e0286714. doi: 10.1371/journal.pone.0286714 (PMC10256181; doi:10.1371/journal.pone.0286714)
Supplement: S1 Appendix — (DOCX) [file pone.0286714.s001.docx]

## S1 Appendix A: Subgroup compared to full cohort

In order to ensure that the subsample (*N* = 892) is representative for the full cohort of 9^th^ grade students allocated to the highest or intermediary school track that were not presented with the measures of interest (*N* = 4.136), both groups have been compared regarding individual student background variables (e.g., gender, SES, language, and migration background) and measures of academic achievement in German, French, and Math. Table A1 shows the distribution of individual student background characteristics.

**Table A1**

*Distribution of Individual Student Background Characteristics in our Subsample and the Reference Group of 9^th^ Grade Students*

|  | **Reference group (*N* = 4.136)** | **Subsample (*N* = 892)** |
| --- | --- | --- |
| **Gender** |  |  |
| Male | 51 % | 54 % |
| Female | 49 % | 46 % |
| **SES** |  |  |
| Low | 17 % | 16 % |
| High | 18 % | 21 % |
| **Language background** |  |  |
| Luxembourgish/German | 46 % | 52 % |
| Other | 53 % | 47 % |
| **Migration background** |  |  |
| Yes | 44 % | 50 % |
| No | 55 % | 50 % |

*Note*. Students in the lowest 25 % of the sample’s SES distribution are considered as having a low SES and students in the highest 25 % as having a high SES. Students are considered as having a migration background when both parents were born outside of Luxembourg, irrespective of the student’s own country of birth. Students are grouped into speaking either Luxembourgish and/or German with at least one of their parents at home or another language (Other). If total does not some up to 100 % for gender, language and migration background, this is due to the share of missings in the data.

Table A2 shows means and standard deviations of academic achievement for both the subsample and the reference group of 9^th^ grade students.

**Table A2**

*Means and Standard Deviations of Academic Achievement in our Subsample and the Reference Group of 9^th^ Grade Students*

|  | **Reference group (*N* = 4.136)** | **Subsample (*N* = 892)** |
| --- | --- | --- |
|  | M (SD) | M (SD) |
| **Academic Achievement** |  |  |
| German | 523.74 (111.51) | 546.48 (108.77) |
| French | 500.01 (103.86) | 514.51 (98.67) |
| Math | 512.93 (94.80) | 531.78 (90.28) |

*Note*. M = Mean. SD = Standard Deviation.

When comparing these two groups, the subsample can be considered as representative for the full cohort of 9^th^ grade students allocated to the highest or intermediary school track in their personal background characteristics and in their academic achievement with the subsample being slightly more alighted with the profile of students attending the highest track (e.g., higher SES, Luxembourgish/German language spoken at home, no migration background and higher academic achievement).
