# Supplementary material for: The relation between Self-Control, Need for Cognition and Action Orientation in secondary school students: A conceptual replication study
Source: PLoS One. 2023 Jun 9;18(6):e0286714. doi: 10.1371/journal.pone.0286714 (PMC10256181; doi:10.1371/journal.pone.0286714)
Supplement: S2 Appendix — (DOCX) [file pone.0286714.s002.docx]

## S2 Appendix B: Descriptive statistics, parcel intercorrelations and covariance matrices

Descriptive statistics (means, standard deviations, range, skewness, and kurtosis) for all the manifest scales are shown in Table A3 and the respective values were computed based on the sum scores of the respective scales and on the sum scores of each parcel (see *Participants and procedure* for details on the computation and handling of missing data).

**Table A3**

*Descriptive Statistics of the Main Variables (Sum Scores) in 9^th^ Grade Students*

|  | *M* | *SD* | Skewness | Kurtosis | Range |
| --- | --- | --- | --- | --- | --- |
| **Scale Sum Scores**  NFC  Trait Self-Control  Effortful Control  Action Orientation${}^{a}$  **Parcel Sum Scores**  NFC P1  NFC P2  NFC P3  NFC P4  Trait Self-Control P1  Trait Self-Control P2  Trait Self-Control P3  Effortful Control P1  Effortful Control P2  Effortful Control P3  Action Orientation P1${}^{b}$  Action Orientation P2${}^{b}$  Action Orientation P3${}^{b}$  Action Orientation P4${}^{b}$ | 35.78  33.32  33.04  11.93  7.50  7.00  8.32  7.54  10.25  10.26  12.81  11.35  10.92  10.76  2.94  3.11  2.97  2.91 | 9.12  6.22  4.97  4.21  2.20  2.12  2.29  2.24  2.28  2.50  2.90  2.17  1.87  2.07  1.42  1.43  1.48  1.45 | 0.05  0.18  0.36  0.01  0.15  0.28  -0.24  -0.03  -0.04  0.12  0.02  0.10  0.33  0.27  -0.00  -0.07  -0.05  0.11 | -0.17  0.06  0.46  -0.12  -0.39  -0.21  -0.47  -0.40  0.11  -0.26  -0.11  0.04  0.72  0.10  -0.59  -0.54  -0.64  -0.53 | 14 – 56  13 – 51  12 – 48  1 – 23  3 – 12  3 – 12  3 – 12  3 – 12  4 – 16  4 – 16  5 – 20  4 – 16  4 – 16  4 – 16  0 – 6  0 – 6  0 – 6  0 – 6 |

*Note. N* = 892. *M* = Mean. *SD* = Standard Deviation. P = Parcel. All parcels were calculated as sum scores of three (e.g., NFC) to six (e.g., AO) items.${}^{a}$ Sum score of all items including both the preoccupation and hesitation dimension of AO (see *3.2* for details). ${}^{b}$ AO parcels including three preoccupation and three hesitation items each (see *3.2* for details). Standard errors for skew and kurtosis are 0.08 and 0.16, respectively.

Reliabilities measured by McDonald’s ω and Cronbach’s α at item and parcel level can be found in Table A4.

**Table A4**

*Reliabilities of the Main Variables in 9^th^ Grade Students*

|  | Level | Cronbach’s α | McDonald’s ω |
| --- | --- | --- | --- |
| NFC  Trait Self-Control  Effortful Control  Action Orientation${}^{a}$ | Items  Parcels  Items  Parcels  Items  Parcels  Items  Parcels | .918  .918  .748  .730  .604  .742  .697  .702 | .919  .918  .749  .738  .530  .744  .703  .703 |

*Note.* ${}^{a}$ All items including both the preoccupation and hesitation dimension of AO.

Intercorrelations between item parcels are display in Table A5.

**Table A5**

*Intercorrelations between Item Parcels in 9^th^ Grade Students*

|  | 1 | 2 | 3 | 4 | 5 | 6 | 7 | 8 | 9 | 10 | 11 | 12 | 13 | 14 |
| --- | --- | --- | --- | --- | --- | --- | --- | --- | --- | --- | --- | --- | --- | --- |
| 1. NFC P1 | - |  |  |  |  |  |  |  |  |  |  |  |  |  |
| 2. NFC P2 | **.79** | - |  |  |  |  |  |  |  |  |  |  |  |  |
| 3. NFC P3 | **.70** | **.67** | - |  |  |  |  |  |  |  |  |  |  |  |
| 4. NFC P4 | **.75** | **.75** | **.77** | - |  |  |  |  |  |  |  |  |  |  |
| 5. TRAIT P1 | **.22** | **.28** | **.22** | **.24** | - |  |  |  |  |  |  |  |  |  |
| 6. TRAIT P2 | **.12** | **.13** | **.08** | **.10** | **.45** | - |  |  |  |  |  |  |  |  |
| 7. TRAIT P3 | **.20** | **.24** | **.20** | **.20** | **.55** | **.44** | - |  |  |  |  |  |  |  |
| 8. EC P1 | **.19** | **.20** | **.20** | **.19** | **.39** | **.43** | **.45** | - |  |  |  |  |  |  |
| 9. EC P2 | **.24** | **.25** | **.25** | **.25** | **.38** | **.34** | **.41** | **.48** | - |  |  |  |  |  |
| 10. EC P3 | **.21** | **.25** | **.25** | **.24** | **.39** | **.37** | **.38** | **.51** | **.49** | - |  |  |  |  |
| 11. AO P1${}^{a}$ | **.21** | **.23** | **.18** | **.20** | **.23** | **.31** | **.27** | **.24** | **.26** | **.29** | - |  |  |  |
| 12. AO P2${}^{a}$ | **.12** | **.13** | **.08** | **.09** | **.17** | **.28** | **.20** | **.21** | **.20** | **.26** | **.38** | - |  |  |
| 13. AO P3${}^{a}$ | **.11** | **.14** | **.10** | **.07** | **.21** | **.29** | **.18** | **.24** | **.16** | **.22** | **.33** | **.39** | - |  |
| 14. AO P4${}^{a}$ | **.07** | **.10** | **.10** | .05 | **.10** | **.25** | **.16** | **.21** | **.12** | **.19** | **.37** | **.40** | **.40** | - |

*Note. N* = 892. TRAIT = Trait Self-Control. EC = Effortful Control. P = Parcel. Parcels were calculated as sum scores of three (e.g., NFC) to six (e.g., AO) items. ${}^{a}$ AO parcels including three preoccupation and three hesitation items each.

*p* ≤ .01 = boldface and underlined. *p* ≤ .05 = boldface.

Latent variable covariance matrices for all four models can be found in Table A6.

**Table A6**

*Latent Variable Covariance Matrices for all four models in 9^th^ Grade Students*

|  | 1 | 2 | 3 | 4 | 5 | 1 | 2 | 3 | 4 | 5 | 6 |
| --- | --- | --- | --- | --- | --- | --- | --- | --- | --- | --- | --- |
| 1. Trait Self-Control | 2.497 |  |  |  |  | 2.537 |  |  |  |  |  |
| 2. Effortful Control | 1.848 | 2.138 |  |  |  | 1.862 | 2.134 |  |  |  |  |
| 3. General Self-Control | 1.735 | 1.735 | 1.628 |  |  | 1.793 | 1.793 | 1.727 |  |  |  |
| 4. NFC | 1.038 | 1.038 | 0.974 | 3.849 |  | 0.531 | 0.531 | 0.511 | 1.000 |  |  |
| 5. Action Orientation | 0.683 | 0.683 | 0.641 | 0.406 | 0.803 | 0.765 | 0.765 | 0.737 | 0.231 | 1.000 |  |
| 6. NFC x Action Orientation | - | - | - | - | - | 0.257 | 0.257 | 0.247 | 0.000 | 0.000 | 1.000 |
|  | 1 | 2 | 3 | 4 | 5 | 1 | 2 | 3 | 4 | 5 | 6 |
| 1. Trait Self-Control | 2.497 |  |  |  |  | 2.503 |  |  |  |  | - |
| 2. Effortful Control | 1.849 | 2.138 |  |  |  | 1.863 | 2.164 |  |  |  | - |
| 3. General Self-Control | 1.735 | 1.735 | 1.628 |  |  | 1.704 | 1.704 | 1.558 |  |  | - |
| 4. NFC | 1.038 | 1.038 | 0.974 | 3.849 |  | 0.472 | 0.472 | 0.431 | 3.842 |  | - |
| 5. Action Orientation | 0.683 | 0.683 | 0.641 | 0.406 | 0.803 | 0.719 | 0.719 | 0.658 | 0.508 | 0.774 | - |

*Note. N* = 892. The Baseline Model (A) is represented in the top left corner. The Moderation Model (B) is represented in the top right corner. The Partial Mediation Model (C) is represented in the bottom left corner. The Complete Mediation Model (D) is represented in the bottom right corner.
