# Supplementary material for: The relation between Self-Control, Need for Cognition and Action Orientation in secondary school students: A conceptual replication study
Source: PLoS One. 2023 Jun 9;18(6):e0286714. doi: 10.1371/journal.pone.0286714 (PMC10256181; doi:10.1371/journal.pone.0286714)
Supplement: Attachment — Submitted filename: Review.pdf [file pone.0286714.s003.pdf]

Comments on PONE-D-22-19185:

This study investigates the influence of need for cognition (NFC) and action orientation (AO) on self-control, focusing on the interplay of NFC and AO. The study is a conceptual replication from earlier research, focusing on 9th graders instead of university students. In line with the original study, the authors found that NFC and AO are correlated predictors of self-control. However, different to the original study they also found that AO moderates the relationship of NFC and AO (stronger influence for action-oriented participants).

The manuscript is well written and methodologically seemingly well done. I only have minor comments, as described in the following:

- I did not completely understand the motivation behind the study. I fully agree that it is important to replicate earlier studies and investigate whether results generalize to different population groups. However, I would appreciate if you could provide more information why you specifically chose 9th graders (compared to university students). Is there any theoretical background, which suggests that there should be differences?
- In my opinion the abstract is quite long and should focus a bit more on the key information of the presented research
- At several points within the manuscript you provide information regarding the mean age of participants (e.g., l. 56). I would recommend to also add information on the standard deviation at this point, to give the reader a better impression how age was distributed within the sample.
- In the Introduction you refer to the delay of gratification study by Mischel et al. (1989) (e.g., l. 80) as well as to the ego-depletion theory by Baumeister (2002) (e.g., l. 210). Even though both studies are highly influential, there is also a lot of critique, especially concerning the replicability of these results. Please add respective information and references.
- I think the Introduction should be shortened at some points. For instance the definition of self-control is repeated at least four times within the manuscript.
- I would appreciate if you could add more information on the procedure of the EpStan (e.g., under which circumstances do the students fill out the questionnaire (at school vs at home)? In which order are questions presented?).
- As you describe within your data analysis section (l. 408), you replaced missing data with corresponding means. Even though this is an established practice to deal with missing data and I assume it is appropriate to do so in the given situation, there are also some problems with this method (e.g., it does not preserve relationships among variables and leads to an underestimation of standard errors). I do not expect you to use a different method, but I would appreciate if you could mention these problems in the manuscript, in order to make the reader aware of possible complications.

To sum up, after addressing the points mentioned above, the manuscript should be a good fit for Plos One. Good luck with your research!
